# Keratoconus corneal architecture after riboflavin/ultraviolet A cross-linking: Ultrastructural studies

**Published:** 2013-07-19

**Authors:** Saeed Akhtar, Turki Almubrad, Iacopo Paladini, Rita Mencucci

**Affiliations:** 1 Cornea Research Chair, Department of Optometry, College of Applied Medical Sciences, King Saud University, Saudi Arabia; 2 Department of Oto-Neuro-Ophthalmology Surgical Sciences, Eye Clinic, University of Florence, Italy

## Abstract

**Purpose:**

Study to investigate the effects of collagen cross-linking on the ultrastructural organization of the corneal stroma in the human keratoconus cornea (KC).

**Methods:**

Three normal, three keratoconus (KC1, KC2, KC3), and three cross-linked keratoconus (CXL1, CXL2, CXL3) corneas were analyzed. The KC corneas were treated with a riboflavin-ultraviolet A (UVA) treatment (CXL) method described by Wollensak et al. Penetrating keratoplasty (PKP) was performed 6 months after treatment. All samples were processed for electron microscopy.

**Results:**

The riboflavin-UVA-treated CXL corneal stroma showed interlacing lamellae in the anterior stroma followed by well-organized parallel running lamellae. The lamellae contained uniformly distributed collagen fibrils (CFs) decorated with normal proteoglycans (PGs). The CF diameter and interfibrillar spacing in the CXL cornea were significantly increased compared to those in the KC cornea. The PG area in the CXL corneas were significantly smaller than the PGs in the KC cornea. The epithelium and Bowman’s layer were also normal. On rare occasions, a thick basement membrane and collagenous pannus were also observed.

**Conclusions:**

Corneal cross-linking leads to modifications of the cornea stroma. The KC corneal structure showed a modification in the CF diameter, interfibrillar spacing, and PG area. This resulted in a more uniform distribution of collagen fibrils, a key feature for corneal transparency.

## Introduction

Keratoconus is a non-inflammatory dystrophy of the cornea, usually bilateral [1]. Typically, the condition starts at puberty, progressing in approximately 20% of cases to such an extent that penetrating keratoplasty becomes necessary [2,3]. This pathology is characterized by axial thinning, fragmentation of the epithelial basement membrane, breaks and scarring at the level of the Bowman’s membrane, keratocyte alteration, and, ultimately, stromal scarring.

In 2003, Wollensak and Spoerl pioneered the corneal riboflavin-ultraviolet A (UVA) cross-linking (CXL) treatment by using photosensitizer riboflavin and UVA light to increase the biomechanical stability of the cornea and resistance to collagenases to inhibit or stop progression of keratoconus [4]. In CXL-treated eyes, maximal keratometry readings and refractive errors remained stable or decreased, whereas visual acuity might improve. Corneal transparency and endothelial cell density remained unchanged by CXL [4-7].

The transparency of the cornea is regulated by the uniform distribution of collagen fibrils (CFs) that are organized into parallel running lamellae [8,9]. The main elements of the collagen fibrils are collagen type 1 and collagen type IV [10,11]. Collagen type VI is also involved in the formation of a microfibrillar structure by lateral aggregation [11,12].

It has been suggested that the uniform distribution of collagen fibrils and lamellar adhesion is maintained by corneal proteoglycans (PGs) [13-15]. The corneal proteoglycans carry a chain of glycosaminoglycans (keratan sulfate or chondroitin sulfate), which are the most abundant negatively charged molecules in the corneal stromal matrix. The corneal proteoglycans lumican, keratocan, and mimecan carry a keratan sulfate chain whereas decorin and biglycan carry a chondroitin sulfate chain. Izzo [16] suggested that decorin and biglycan might not play a significant role in corneal transparency because the binding of dermatan sulfate small leucine-rich proteoglycans (SLRPs; decorin and biglycan) occurs at the d and e bands of collagen, in contrast to the keratan sulfate SLRPs (lumican, keratocan, and mimecan) that bind to the a and c bands of collagen [17]. Experiments suggest that the protein core on the keratan sulfate proteoglycan (KS-PG) regulate the diameter of the collagen ﬁbrils [18-20] whereas the sulfated glycosaminoglycans (the GAG chain) maintain interfibrillar spacing by extending outward from the protein core [16].

Most studies on collagen cross-linking treatment of keratoconic progression suggest that collagen cross-linking treatment through riboflavin-sensitized UVA irradiation increases corneal rigidity [21,22]. McCall et al. [23] explained the mechanism of collagen cross-linking with riboflavin-UVA, in which riboflavin-sensitized UVA irradiation generates free radicals and reactive oxygen species such as superoxide anion (O_2_), hydroxyl radical (OH), and hydrogen peroxide (H_2_O_2_). This mainly occurs by the type I pathway of photosensitized oxidation.

Few studies have demonstrated that the CXL treatment increases the diameter of the collagen fibril in the cornea and sclera [24-26]. Wollensak et al. [24] investigated the collagen fibril diameter in the rabbit cornea after riboflavin-UVA treatment. The study revealed that the collagen fibril diameter increased in the anterior stroma and posterior stroma of the treated cornea compared to the collagen fibril diameter of the normal corneal stroma. The diameter of the CF in the anterior stroma of the treated cornea was also significantly higher compared to the CF diameter of the posterior stroma of the same cornea. Choi et al. [26] investigated the effect of riboflavin-UVA treatment on the corneoscleral strips from the donor tissue by using the histology, mechanical measurement, scanning electron microscopy, and atomic force microscopy. Their study showed that the treatment led to structural improvements in the corneal tissue as well as increases in thickness (107%), fibril density (112%), area (110%), diameter (103%), and parallel arrangement.

Mencucci et al. [25] assessed the effect of riboflavin-UVA treatment on the keratocyte population of the CXL cornea by using K_i_-67 and CD34. The authors reported that initially the CXL treatment led to keratocyte damage, but 6 months after the treatment, repopulation of keratocytes was observed. The keratocyte population in the CXL cornea was similar to the keratocyte population in the normal cornea.

Literature on ultrastructural studies of CXL cornea is scant. In the present study, we assessed the effect of CXL treatment on the ultrastructural organization of the collagen fibrils and proteoglycans of the keratoconus cornea.

## Methods

### Patient details

Tissue procurement and use were in accordance with the Declaration of Helsinki and local regulations. The study was ethically approved by the Local Ethical Committee of King Saud University, Saudi Arabia.

Three sets of human corneal buttons were used for the present study. Group 1 included three normal corneas (32, 37, 39 years old) from the Eye-Bank of Lucca used as the control sample. Group 2 included three corneal buttons with keratoconus corneas (33, 36, 37 years old) without sub-epithelial scarring removed after PKP was performed. Group 3 included three keratoconus corneal buttons, which came from three patients aged 58 (CXL1), 37 (CXL2), and 21 (CXL3) years old who had been treated with cross-linking 6 months before, using the Wollensak et al. [4] technique. These were removed because of poor vision.

The 58-year-old patient (CXL1) and the 37-year-old patient (CXL2) had a full thickness graft. All the lamellae in the anterior, middle, and posterior were present in the sample. The 21-year-old patient (CXL3) had undergone a lamellar graft. Only the anterior and middle lamellae were present in the sample.

All the keratoconus donors gave permission for their tissue to be used for research. In groups 2 and 3, all keratoconus corneas were in Krumeich stage 3. The Amsler– Krumeich classification, based on patients’ refraction, mean central K-reading, corneal signs, and corneal thickness, was used for keratoconus grading. The patients were informed about the severe deterioration of their keratoconus and gave their permission to perform corneal cross-linking before PKP. The corneas used in this study had not been exposed to steroid therapy or undergone surgical treatment. Antibiotic drops were used 5 days after cross-linking until the new epithelium had formed completely. Clinically, the corneas were not normal and were removed. Immediately after the corneas were removed, the cornea buttons were processed for electron microscopy.

### Processing method

The central parts of the corneal buttons were studied with an electron microscope. Each corneal button was divided into two parts. The first part was processed to study the distribution of proteoglycans throughout the cornea. The corneal part was further divided into 1 mm^2^ pieces and fixed in 2.5% glutaraldehyde (TAAB Laboratories Equipment Ltd, Reading, UK) containing 0.05% Cuprolinic Blue (BDH, Dorset, England) using a critical electrolyte concentration mode in 25 mM sodium acetate (TAAB Laboratories Equipment Ltd) with 0.1 M magnesium chloride acetate (TAAB Laboratories Equipment Ltd) buffer overnight at room temperature. The tissue was then dehydrated through a graded ethanol series (50% to 100%) and 100% acetone. The tissue was immersed in a mixture of acetone and TAAB 031 resin for 8 h. The tissue was further immersed in 100% TAAB 031 resin for 8 h (three times) and polymerized in TAAB 031 resin for 12 h at 60 °C.

The second part was processed to study the morphology and diameter of the cornea. The corneal part was further divided into 1 mm^2^ pieces and immersed in 2.5% glutaraldehyde in 0.1 M phosphate buffer (PBS; 0.1M Na_2_HPO_4_+ NaH_2_PO_4_) for 2 h and post-fixed in 1% osmium tetroxide in 0.1 M phosphate buffer for a further hour at room temperature. The tissue was then dehydrated through a graded ethanol series (50% to 100%) and 100% ethanol. They were immersed in the mixture of acetone and TAAB 031 resin for 8 h. The tissues were further immersed in 100% TAAB 031 resin for 8 h (three times) and polymerized in TAAB 031 resin for 12 h at 60 °C.

All the blocks contained 1 mm^2^ pieces of tissue. One block from each cornea was used because the CXL corneas tissues were small. Three corneas in each treatment were cut to get ultrathin sections. These sections were collected on 200 mesh copper grids. The ultrathin sections were stained with 2% uranyl acetate and 2% lead citrate. The sections were observed with transmission electron microscopy (Jeol 1400; Jeol, Akishima, Japan). Three sections from each block and two images from each section were used for the quantitative analysis. The smallest diameter across the cross section of the collagen fibril was the minimum diameter. The minimum diameter of each ﬁbril was measured rather than an average value to avoid errors caused by any obliqueness in ﬁbril cross sections. The minimum diameter and interfibrillar spacing were measured using soft imaging analysis software (iTEM soft Imaging Systems GmbH, Münster, Germany). The PG analysis was performed from the sections of the Cuprolinic Blue fixed tissue blocks. The sections were not stained with uranyl acetate and lead citrate to visualize PGs. The images of the collagen fibrils and proteoglycans were taken from the parallel running lamellae below the interlacing lamellae in the anterior stroma. The Mann–Whitney U test (SPSS Inc., Chicago, IL) was used for statistical analysis because the data were not normally distributed. Graphs were drawn by using the SPSS program.

## Results

### Ultrastructure of keratoconus and cross-linked keratoconus corneas

**Figure 1:**
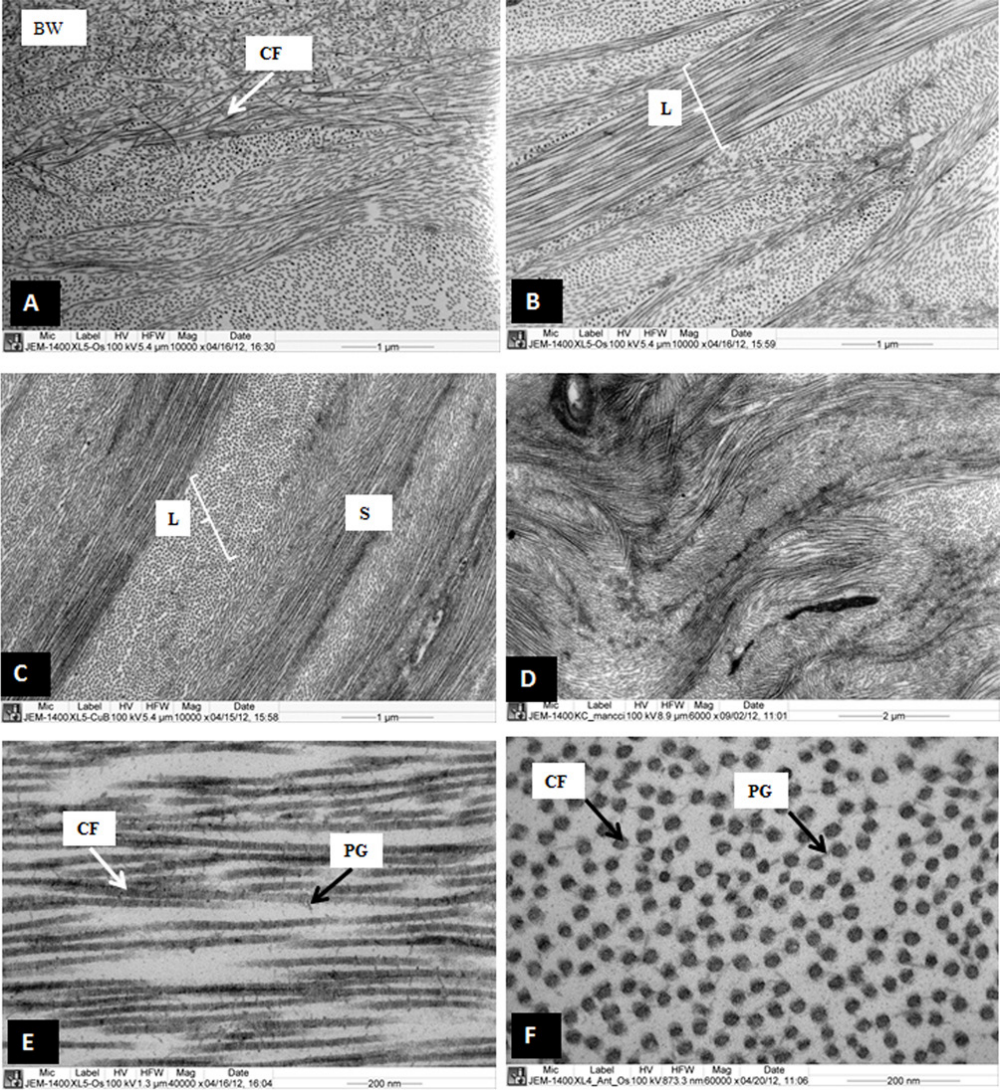
Electron micrograph of cross-linked sample 1 and keratoconus corneas. **A**: Collagen fibrils are emerging from the posterior part of the Bowman’s layer. **B**: Interlacing lamellae are present in the anterior stroma. **C**: Parallel running collagen fibril lamellae are present in the posterior part of the anterior stroma. **D**: Part of the keratoconus (KC) corneas are showing undulating lamellae. **E**: Longitudinally running collagen fibrils are decorated with proteoglycan (PGs), in the posterior part of anterior stroma. **F**: Cross section of the collagen fibrils are decorated with PGs in the anterior part of the stroma. BW= Bowman’s layer, CF=Collagen fibrils, L=Lamella, PG=Proteoglycans, S=Stroma.

**Figure 2:**
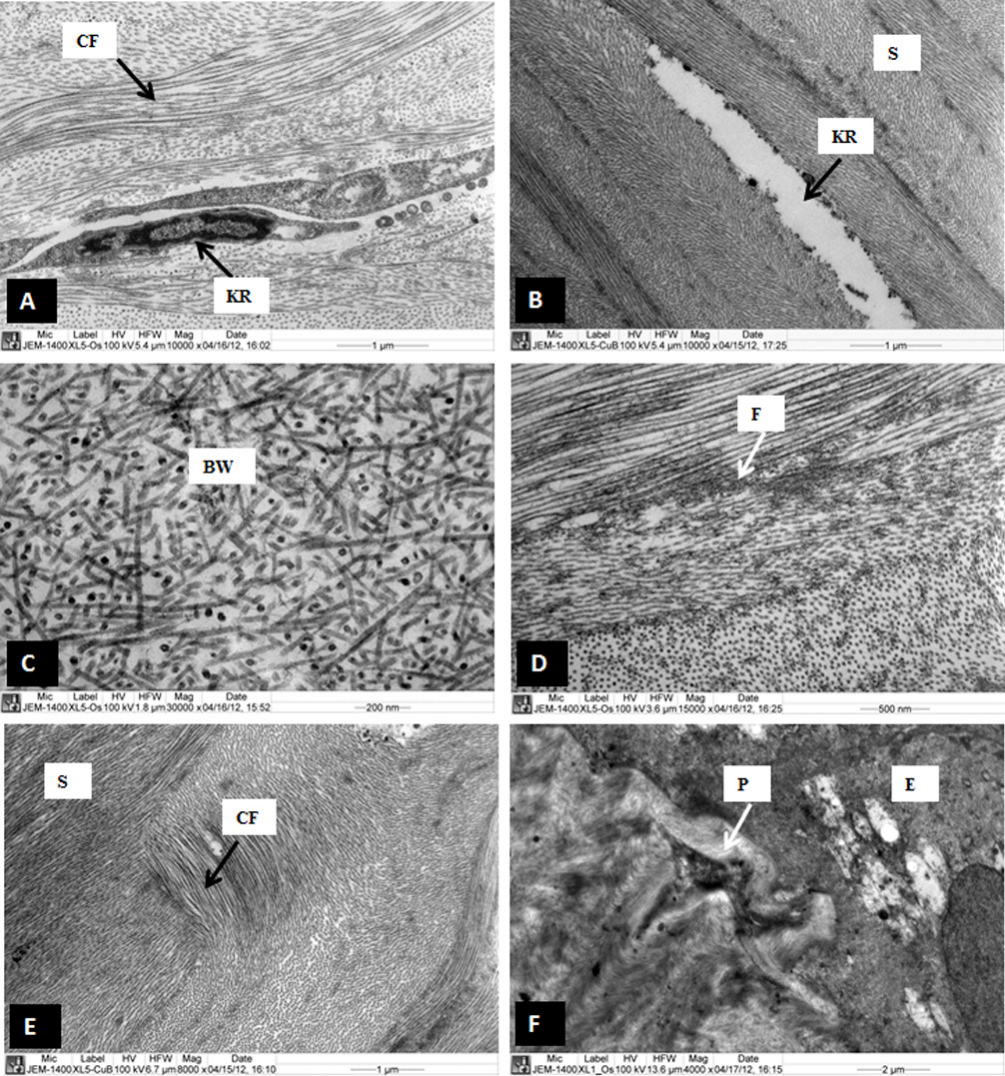
Electron micrograph of sample cross-linked (CXL) samples 1, 2, and 3. **A**: A normal keratocyte with large nucleus is present in the anterior part of the stroma of CXL1. **B**: An electron lucent part of keratocyte is present in the anterior part of the stroma of CXL3. **C**: A part of Bowman’s layer which appeared normal in CXL1. **D**: Patches of microfilaments are present in the anterior stroma of CXL1. **E**: Randomly running collagen fibrils are present in the anterior stroma in the anterior stroma of CXL1. **F**: A collagenous pannus at sub-epithelial region is pushing basal epithelial cell in CXL2. E=Epithelium, BW=Bowman’s layer, CF=Collagen fibrils, F=Micro-filaments, KR=Keratocyte, p=Pannus, S=Stroma.

The CXL treatment improved the organization of the lamellar structure of the CXL corneas (CXL1, CXL2, CXL3). Collagen fibrils emerged from the posterior end of the Bowman’s layer (BW) in the sub-Bowman’s layer region (Figure 1A). The anterior stroma contained interlacing lamellae (Figure 1B). Below the interlacing lamellae and in the middle stroma, the lamellae ran parallel to each other (Figure 1C) instead of undulating as the lamellae in the KC cornea (Figure 1D). The collagen fibrils in the lamellae of the CXL cornea ran parallel to one another and were decorated with proteoglycans (Figure 1E). The distribution of the collagen fibrils, diameter, and interfibrillar spacing was improved by the CXL treatment. The cross section of the collagen fibrils showed that the collagen fibrils were uniformly distributed and connected to each other by PG filaments (Figure 1F). The keratocytes in the anterior stroma contained a large nucleus, a prominent endoplasmic reticulum, and all other cell organelles (Figure 2A). Occasionally in the CXL3 cornea, the anterior stroma contained keratocytes that lacked a nucleus and cell organelles (Figure 2B). These keratocytes appeared as an electron lucent space containing electron-dense material at the peripheral region. The electron-dense material might be remnants of cell organelles.

The epithelium, basement membrane, hemidesmosomes, and Bowman’s layer were not intended to be influenced by the CXL treatment. The epithelium and Bowman’s layer of the CXL corneas were similar to the epithelium and Bowman’s layer of the normal cornea (Figure 2C). The major part of the CXL corneas observed under an electron microscope contained normal hemidesmosomes and basement membrane. Occasionally, degenerate hemidesmosomes and a thick basement membrane were observed in some places in the stroma in the CXL1 cornea (not shown). Patches of microfilaments were present in some places in the stroma (Figure 2D). Occasionally, a collagenous pannus was seen in the CXL2 cornea, and curved collagen fibrils were observed in the CXL3 cornea (Figure 2E,F). These collagen fibrils could be a remnant of the undulating lamellae, which presumably became parallel after the riboflavin-UVA treatment.

### Mean collagen fibril diameter, interfibrillar spacing and density of normal corneas, keratoconus corneas, and cross-linked keratoconus corneas

**Figure 3:**
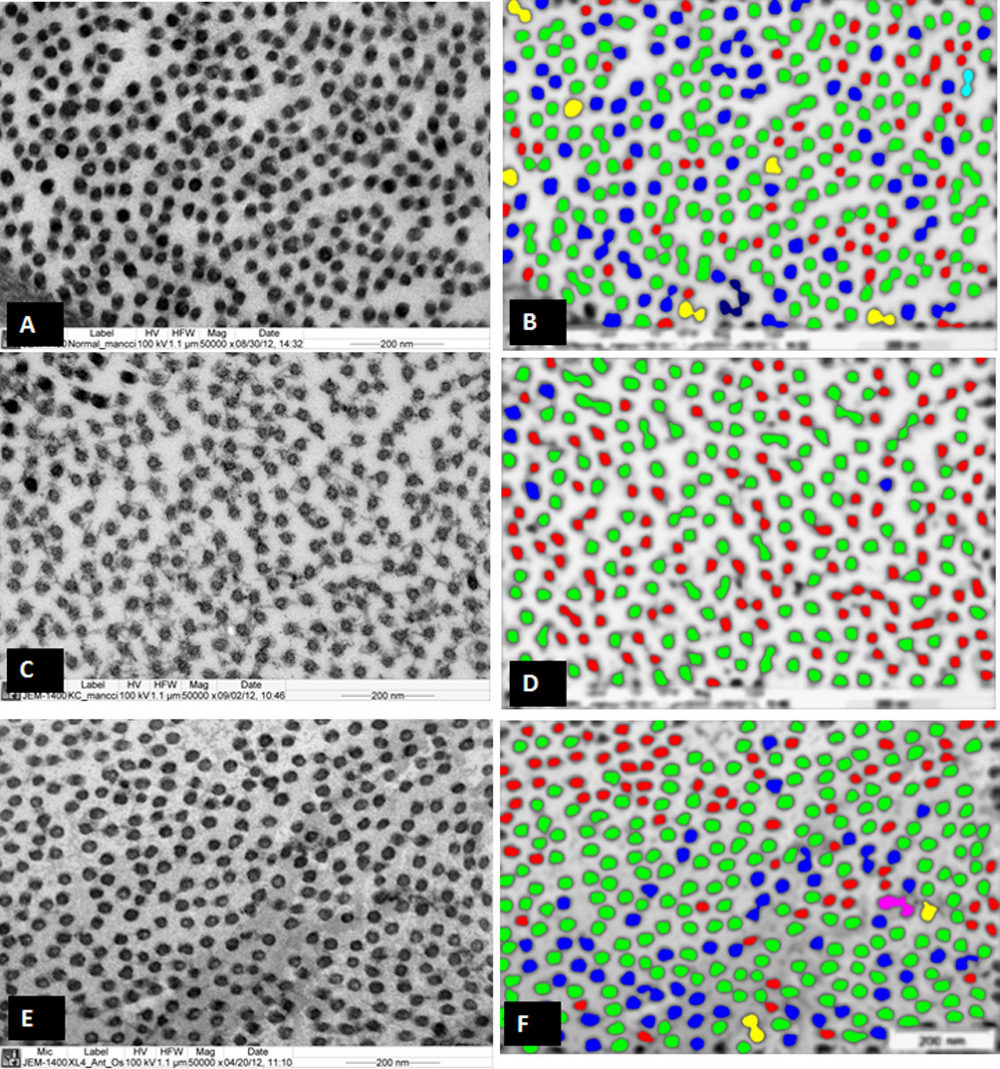
Electron micrograph and digital images of the collagen fibrils of normal, keratoconus (KC), and cross-linked (CXL) corneas. **A**: Electron micrograph of collagen fibrils which are present in the normal human corneas. **B**: Digital image obtained after processing the image shown in **A**. **C**: Electron micrograph of the collagen fibrils which are present in the KC corneas. **D**: Digital image is obtained after processing the image shown in **C**. **E**: Electron micrograph of collagen fibrils which are present in the CXL corneas. **F**: Digital image obtained after processing the image shown in **E**. The images were displayed by using color coding to demonstrate the distribution of the variable diameters of collagen fibrils. Collagen fibril color coding: Red=10–15 nm, Green=15–20 nm, Blue=20–25 nm, Yellow=24–30 nm, Aqua=30–35 nm, Pink=35–40 nm, Brown=40–45 nm.

The ultrastructural images of the CFs were processed with iTEM analysis software and color coded to demonstrate the distribution of the CF according to their size as described previously [27]. The ultrastructural images of normal corneas (Figure 3A), keratoconus corneas (Figure 3C), and CXL-treated corneas (Figure 3E) were color coded and are shown in Figure 3B,D,F, respectively.

**Figure 4:**
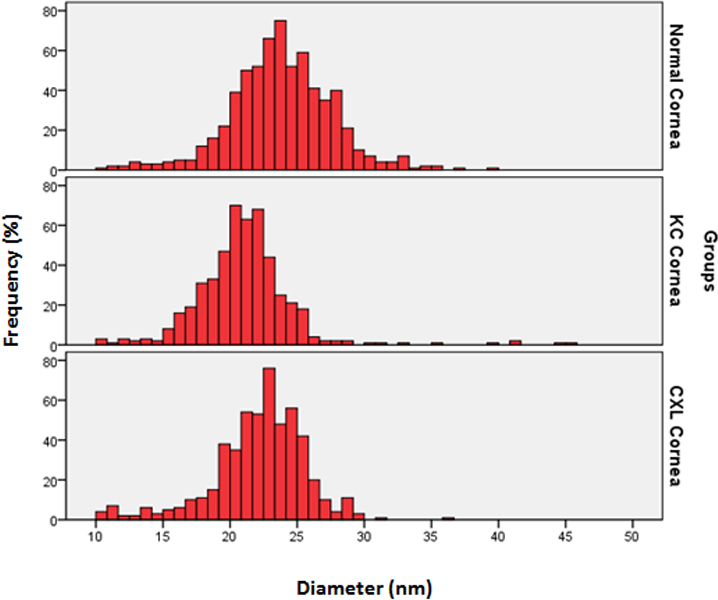
Distribution of collagen fibril diameter (nm) in normal corneas, keratoconus (KC) corneas, and cross-linked (CXL) corneas. The data were derived from three corneas and six images of each group.

The mean CF diameters of the normal corneas, keratoconus corneas, and CXL corneas are shown in Table 1. The CF diameter of the CXL cornea was significantly (p<0.001) larger compared to the CF diameter of the KC cornea, which suggests that the riboflavin-UVA treatment increases the CF diameter of the KC cornea. The CF diameter of the normal cornea was significantly larger compared to those in the KC and CXL corneas. The graph presented in Figure 4 shows that most of the collagen fibril diameter in the CXL cornea and the normal cornea were distributed in the range of 20–30 nm whereas the CF diameters in the KC cornea were distributed in the range of 15–25 nm. This suggests that the riboflavin-UVA treatment increases the diameters of the KC corneas and brings them closer to the normal corneal collagen fibril diameter.

**Table 1:** Mean diameter, inter-fibrillar spacing and density of collagen fibrils of normal, keratoconus and CXL cornea.

Sample	Number of cornea	Number of collagen fibrils	Mean Diameter	Median Diameter	Inter-fibrillar spacing
Normal Cornea	3	648	23.8±4.5	23.74	38.0±7.8
Keratoconus Cornea	3	496	21.3±2.5	20.99	31.3±3.4
XL Cornea	3	523	22.4±3.1	22.57	38.9±6.4

Significant difference between Normal and KC p<0.0001 Significant difference between Normal and CXL p<0.0001 Significant difference between KC and CXL p<0.0001

The interfibrillar spacing of the normal, KC, and CXL corneas is shown in Table 1. The interfibrillar spacing in the CXL cornea was significantly higher (p<0.0001) compared to the interfibrillar spacing of the KC cornea. There was no significant difference between the interfibrillar spacing of the normal corneas and that of the CXL corneas. This suggested that the interfibrillar spacing was altered by the CXL treatment. The spacing changes observed in the CXL cornea could be related to post-operative edema of the corneas. Due to the small sample size, generalizations cannot be made.

### Mean proteoglycan area and density of normal corneas, keratoconus corneas, and cross-linked keratoconus corneas

**Figure 5:**
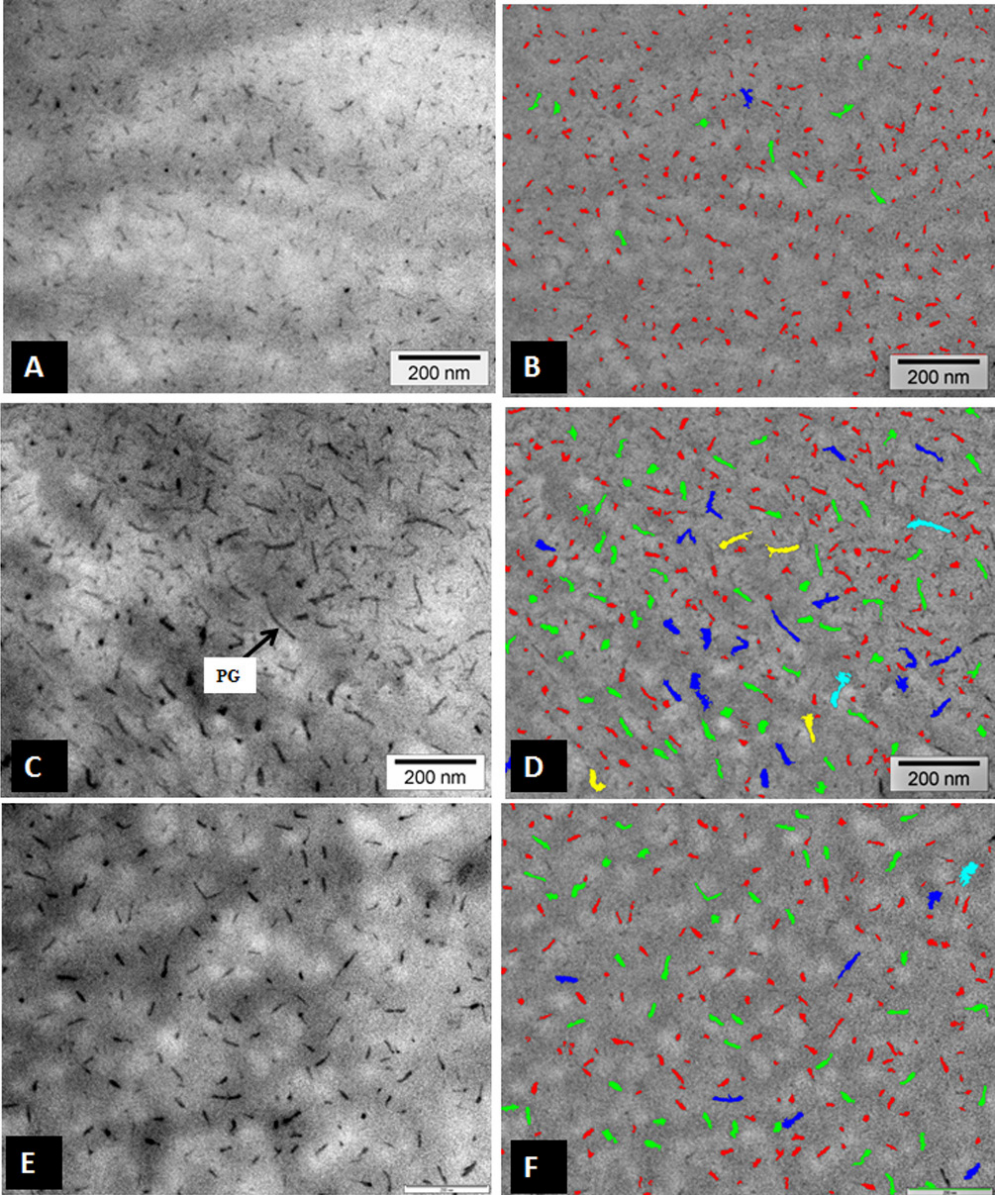
Electron micrograph and digital images of proteoglycans (PG) of normal, keratoconus (KC), and cross-linked (CXL) corneas. **A**: Electron micrograph of PGs which are present of normal corneas. **B**: Digital image is obtained after processing image shown in **A**, showing variable area distribution of PGs. **C**: Electron micrograph which are present in PGs of KC corneas. **D**: Digital image is obtained after processing image shown in **C**, showing variable area distribution of PGs. **E**: Electron micrograph of PGs of CXL corneas. **F**: Digital image obtained after processing image shown in **E**, showing variable area distribution of PGs. PG=Proteoglycan Proteoglycans color coding: Red=50–350 nm^2^, Green=350–650 nm^2^, Blue=650–950 nm^2^, Yellow=950–1250 nm^2^, Aqua=1250–1550 nm^2^, Pink=1550–1850 nm^2^, Brown=1850–2150 nm^2^.

Similar to the CF image analysis, digital image analysis of PGs was performed with iTEM analysis software and color coded to demonstrate the distribution of the PGs according to their size as described previously [27]. The electron micrographs and digital images are shown in Figure 5. The ultrastructural images of the normal PGs (Figure 5A), keratoconus corneal PGs (Figure 5C), and CXL-treated corneal PGs (Figure 5E) are color coded in Figure 5B,D,F respectively.

**Figure 6:**
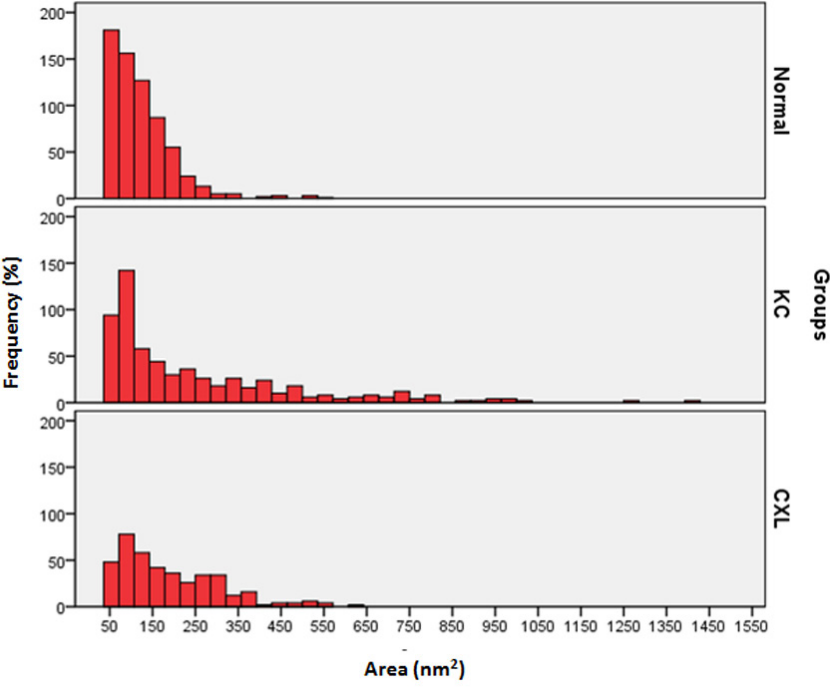
Distribution of proteoglycan area (nm^2^) in normal, keratoconus, and cross-linked corneas. The data were derived from three corneas and six images of each group.

The PG areas of the normal corneas, KC corneas, and CXL corneas and their density are shown in Table 2. The PG area of the CXL corneas was significantly smaller compared to the PG area of the KC corneas. The PG area of the normal corneas was smaller compared to the PG area of the KC corneas and the CXL corneas. The graph presented in Figure 6 shows the distribution of PGs in the normal, KC, and CXL corneas. The largest PG areas in the normal, KC, and CXL corneal stroma were 514.95 nm^2^, 1420.95 nm^2^, and 650.62 nm^2^, respectively (Figure 6). The density of the PGs in the CXL corneas (209/mµ^2^) was also significantly reduced compared to that of the KC corneas (311/ mµ^2^).

**Table 2:** Mean area and density of PGs of normal, keratoconus, and XL cornea.

Sample	Number of corneas	Number of PGs	Mean PGs Area (nm^2^) ±SD	Median Area (nm^2^)
Normal Cornea	3	662	122.26±78	104.22
Keratoconus Cornea	3	622	252.3±135	157.00
XL Cornea	3	631	189.0±149	156.98

Significant difference between Normal and CKC p<0.0001 Significant difference between Normal and CXL p<0.0001 Significant difference between KC and CXL p<0.0001.

## Discussion

In the present study, the riboflavin-UVA treatment showed a positive impact on all the samples. The riboflavin-UVA-treated cornea showed interlacing lamellae in the anterior stroma just below the Bowman’s layer. Below the interlacing lamellae, well-organized parallel running lamellae were observed. The interlacing lamellae and parallel running lamellae contained uniformly distributed collagen fibrils. The collagen fibrils in the lamellae ran parallel to one another and were decorated with normal proteoglycans. The diameter of the collagen fibrils of the riboflavin-UVA-treated corneas was increased compared to the collagen fibril diameter of the KC corneas. Patches of the microfilaments were rarely observed in the CXL corneal stroma. Before the treatment, the corneas were diagnosed as KC corneas. The riboflavin-UVA treatment changed the KC features into normal corneal features in the CXL corneas.

In all corneas (CXL1, CXL2, and CXL3), the keratocytes had normal cell organelles, but in the CXL3 patients, occasionally the keratocytes did not have a nucleus and cell organelles. UVA might have caused degeneration of the keratocytes as has been reported previously in clinical studies [28]. The keratocytes might have degenerated due to apoptosis, and the electron-dense material could be remnants of the cell organelles. Regeneration and proliferation of keratocytes 6 months after the riboflavin-UVA treatment were observed in the CXL corneas [25]. The authors suggested that the cross-linking treatment might have stimulated a distinct biologic response to enhance the keratocyte population [25]. In contrast to the previous authors [25], Messmer et al. [29] observed a significant reduction in keratocyte counts in the entire cornea in the riboflavin-UVA corneas compared with the keratoconus and normal corneas.

In the CXL corneas, occasionally, a few randomly running collagen fibrils were present in the parallel running lamellae below the interlacing lamellae in the anterior stroma. These curved collagen fibrils could be the remnants of undulating lamellae, which presumably became parallel after the riboflavin-UVA treatment. The randomly running collagen fibrils may have been present in the normal corneas. To our knowledge, no randomly running collagen fibrils have been observed in normal corneas. The feature was observed only on rare occasions in the CXL corneas. Based on this rare occurrence of curved collagen fibrils, generalizations cannot be made from the present study. The transformation of the keratoconic corneal stroma into a normal corneal stroma suggested that the treatment was successful.

The riboflavin-UVA treatment does not affect the hemidesmosomes, basement membrane, and Bowman’s layer. This could be the reason that the degenerate hemidesmosomes and thick basement were present in the treated cornea. The presence of these degenerate hemidesmosomes and thick basement suggest that the riboflavin-UVA treated corneas were KC corneas before the treatment. The presence of collagenous pannus and curved collagen fibrils in samples CXL 2 and CXL3, respectively, suggested that the treatment might not be effective in those regions.

There is a strong relationship between collagen fibril formation and PG core proteins, i.e., lumican, keratocan, mimecan, and decorin [16,30]. Chakravarti et al. [30] demonstrated that the lumican-deﬁcient posterior stroma displayed a pronounced increase in ﬁbril diameter, large ﬁbril aggregates, altered ﬁbril packing, and poor lamellar organization. It was also revealed that there was a 25% reduction in the KS content of whole eyes in the lumican-deﬁcient mice stroma. Izzo [16] suggested that the corneal clouding observed in lumican-deficient mice may be multifactorial: abnormal fibril assembly, lateral fusion caused by the lack of a lumican protein core, and altered interfibrillar spacing because of the lack of lumican-bound keratan sulfate. Zhang et al. [31] investigated the effect of riboflavin-UVA treatment specifically on the interaction of collagen and PGs in an in vitro and an ex vivo model reaction system. Zhang et al. [31] demonstrated that cross-linking keratocan, lumican, mimecan, and decorin core proteins to collagen type 1 was observed when these core proteins were present in the native glycosylated form. The presence of the KS chains could affect the conformation of these two core proteins as they bind to collagen (enhancing their riboflavin-UVA treatment cross-linking to collagen).

Our results showed that the CF diameter in the CXL corneas was significantly smaller compared to the CF diameter of the normal corneas. The CF diameter in the CXL corneas was significantly larger compared to the CF diameter of the KC corneas suggesting that CXL treatment increased the CF diameter. In addition, the interfibrillar spacing of the CXL corneas increased and became approximately similar to the interfibrillar spacing of the normal corneas. Our PG analysis showed that the PG area was reduced in the CXL corneas compared to that in the KC corneas. This suggests that CXL treatment normalizes not only collagen fibril synthesis but also PG synthesis. We believe that CXL treatment might have multifactorial effects on collagen fibril organization. In the CXL corneas, the normal assembly and lateral and linear fusion could be due to cross-linking of native glycosylated core proteins with collagen type 1, and uniform interfibrillar spacing could be due to cross-linking in core-protein bound keratan sulfate. Due to the small size of the sample, generalizations cannot be made.

Collagen cross-linking with riboflavin-UVA treatment significantly increases the collagen fibril diameter, which leads to the significant increase in the biochemical strength (70−300%) of the collagen fibrils [32,33]. Parry [34] reported that the ultimate tensile strength (UTS) of tissues containing type I collagen has been correlated with the ﬁbril diameter. Contradictory to Perry [34], Silver et al. [35] reported that during self-assembly of collagen type 1, the mechanical properties of collagen ﬁbrils are more dependent on collagen ﬁbril length than diameter.

Christiansen et al. [36] studied the relationship between the assembly of collagen type 1 and its mechanical properties. The authors suggested that self-assembly of collagen type I mediated by the formation of ﬁbrillar mediate subunits (approximately 4 nm) laterally and linearly fuse resulting in the formation of ﬁbrillar subunit growth. Lateral fusion of ﬁbrillar subunits increases the low strain resistance to deformation but does not strongly affect high strain stiffness and UTS. Lateral fusion appears to be promoted by electrostatic interactions. However, the longitudinal fusion of fibrillar subunits (i.e., linear growth) increase the high strain resistance to deformation and UTS but does not affect low strain stiffness. In our studies, the CXL treatment transformed the undulating or curved collagen fibrils of the keratoconus corneas into linear or parallel running collagen fibrils, which presumably provide UTS to the collagen fibrils. At the same time, the increase in the collagen fibril diameter may have increased the low strain resistance to deformation of the cornea.

The clinical and structural analysis of the cross-linked corneas suggests that cross-linking treatment transforms the architecture of the stroma into that of a normal stroma. Riboflavin-UVA treatment induces resistance to proteolytic enzymes such as collagenase [37] and synthesizes large collagen molecular aggregates [38] followed by cross-linking of core proteins to collagen type 1. The riboflavin-UVA treatment may increase corneal stiffness because of the additional covalent binding between the collagen molecules [26]. The structural changes in the KC corneas could be caused by hydration rather than by cross-linking during riboflavin-UVA treatment [39].

To make generalizations, a large number of samples are required to carry out further studies that measures changes in the diameter of collagen fibrils, interfibrillar spacing, and PG distribution. However, uniform distribution of the collagen fibrils suggests that cross-linking treatment changed the abnormal keratoconic CF distribution into normal CF distribution. The keratocytes degenerated during the initial stages of treatment, but these keratocytes were regenerated in the later stages of the development of the stroma.
